# The Effect of IFN-γ, Alum and Complete Freund Adjuvant on TNP-KLH Induced Ig.G_1_, IgE and IgG_2a_ Responses in Mice

**DOI:** 10.1155/S0962935194000542

**Published:** 1994

**Authors:** R. van Ommen, A. E. C. M. Vredendaal, M. de Gooyer, A. van Oudenaren, H. F. J. Savelkoul

**Affiliations:** Department of Immunology Erasmus University PO Box 1738 Rotterdam 3000 DR The Netherlands

## Abstract

Adjuvants are considered to play an important role in directing the isotype and amount of antibodies produced upon immunization by conducting the development of either Th-1 or Th-2 cells upon T-cell stimulation. This is based on the different cytokine production patterns that were observed after *in vitro* resttmulation of T cells isolated from mice immunized with antigen either adsorbed on alum or emulsified in complete Freund adjuvant (CFA). However, other studies suggest that primarily the type of antigen determines which isotypes are produced and to what extent. In these studies, however, IgE was not determined. Therefore, this study examined whether alum and CFA influenced the amount and/or ratio of IgG_1_, IgE and IgG_2a_ produced after TNP-KLH immunization. Similar levels of IgG_1_, IgE and IgG_2a_ antibodies were found upon immunization with TNP-KLH either adsorbed on alum or emulsified in CFA. Moreover, administration of IFN-γ in combination with TNP-KLH adsorbed on alum did not increase the amount of IgG_2a_ produced. IFN-γ treatment resulted in an increased IL-6 and decreased IFN-γ production by spleen cells upon Con A stimulation, whereas it did not change the IL-4 production in similar conditions. The presented results suggest that upon immunization with TNP-KLH high IL-4 levels are produced, resulting in an antibody response that is dominated by IgG_1_, independent of the adjuvant employed. The IL-4 inducing property of TNP-KLH is substantiated by the finding that repeated immunization of mice with TNP-KI, without adjuvant, increases the serum total IgE level. The presented data suggest that the carrier part of TNP-KLH preferentially results in Th-2 cell activity after which the adjuvant merely enhances the antibody responses generated.


Research Paper

Mediators of Inflammation 3, 387–392 (1994)

### The effect of IFN-γ, alum and complete Freund adjuvant on TNP–KLH induced $IgG_1$, IgE and $IgG_{2a}$ responses in mice

**R. van Ommen,**[CA] **A. E. C. M. Vredendaal, M. de Gooyer, A. van Oudenaren, and H. F. J. Savelkoul**

Department of Immunology, Erasmus University, PO Box 1738, 3000 DR Rotterdam, The Netherlands

[CA] Corresponding Author

**ADJUVANTS are considered to play an important role in directing the isotype and amount of antibodies produced upon immunization by conducting the development of either Th-1 or Th-2 cells upon T-cell stimulation. This is based on the different cytokine production patterns that were observed after *in vitro* restimulation of T cells isolated from mice immunized with antigen either adsorbed on alum or emulsified in complete Freund adjuvant (CFA). However, other studies suggest that primarily the type of antigen determines which isotypes are produced and to what extent. In these studies, however, IgE was not determined. Therefore, this study examined whether alum and CFA influenced the amount and/or ratio of $IgG_1$, IgE and $IgG_{2a}$ produced after TNP–KLH immunization. Similar levels of $IgG_1$, IgE and $IgG_{2a}$ antibodies were found upon immunization with TNP–KLH either adsorbed on alum or emulsified in CFA. Moreover, administration of IFN-γ in combination with TNP–KLH adsorbed on alum did not increase the amount of $IgG_{2a}$ produced. IFN-γ treatment resulted in an increased IL-6 and decreased IFN-γ production by spleen cells upon Con A stimulation, whereas it did not change the IL-4 production in similar conditions. The presented results suggest that upon immunization with TNP–KLH high IL-4 levels are produced, resulting in an antibody response that is dominated by $IgG_1$, independent of the adjuvant employed. The IL-4 inducing property of TNP–KLH is substantiated by the finding that repeated immunization of mice with TNP–KLH, without adjuvant, increases the serum total IgE level. The presented data suggest that the carrier part of TNP–KLH preferentially results in Th-2 cell activity after which the adjuvant merely enhances the antibody responses generated.**

**Key words:** Adjuvant, B-cell memory, $IgG_1$, IgE and $IgG_{2a}$ responses

### Introduction

Helper T cells (Th) play an important role in antigen induced humoral immune responses.[1] At least two effector subpopulations of murine Th cells, differentiated according to the spectrum of cytokines produced, have been reported.[2,3] Th-1 cells exclusively produce IL-2 and IFN-γ, whereas Th-2 cells exclusively produce IL-4, IL-5 and IL-10, but not IL-2 and IFN-γ.[3] It has been described that adjuvant, used to facilitate the induction of antigen specific immune responses, plays an important role in determining which T cell subset will be activated *in vivo*.[4,5] Immunization of mice with either ovalbumin (OVA) or ragweed pollen extract in the presence of alum resulted in T cells that produced IL-2, IL-4 and IFN-γ upon *in vitro* restimulation with the same antigen. In contrast, immunization with either OVA or ragweed pollen extract in the presence of complete Freund adjuvant (CFA) resulted in T cells that produced IL-2 and IFN-γ, but not IL-4 upon *in vitro* restimulation.[6] The amount of IFN-γ produced by T cells from mice primed with antigen emulsified in CFA and cultured in the absence of antigen was markedly higher than the production of IFN-γ in similar cultures of T cells from mice primed with antigen adsorbed on alum.[6]

Based on these results is was suggested that alum augments preferentially Th-2 mediated responses, whereas CFA augments preferentially Th-1 mediated responses. Antigen adsorbed on alum would thus be expected to induce $IgG_1$ and IgE responses, since IL-4 is involved in the process of isotype switching to $IgG_1$ and IgE.[7,8] Upon immunization in the presence of CFA, the same antigen would be expected to elicit a preferential $IgG_{2a}$ response, since IFN-γ has been reported to enhance $IgG_{2a}$ production. Moreover, IL-4 inhibits $IgG_{2a}$ production, whereas IFN-γ decreases both the $IgG_1$ and IgE synthesis,[9] indicating that the balance between these two cytokines determines the

© 1994 Rapid Communications of Oxford Ltd amount and isotype of antibodies that will be produced.

The authors have recently shown that primary TNP specific IgE responses are completely dependent on IL-4 and can be largely inhibited by IFN-γ, whereas secondary antigen specific IgE responses are partially IL-4 independent and can hardly be inhibited by IFN-γ in a concentration that inhibits the primary TNP specific IgE response.[10]

In the literature contradictory results have been described concerning the effects on the isotype production profile and concentration of antibody depending on the type of adjuvant employed. Moreover, immunization with different antigens (HSA, FITC, cytochrome C) showed different effects on the isotype production profile and amount of antibodies when administered either adsorbed on alum or emulsified in CFA.[11–13] However, in these studies the IgE isotype was not determined. The presence of this isotype, however, is indicative for the balance between IL-4 and IFN-γ *in vivo*.

It was reported by Beck *et al.* that immunization with OVA in alum resulted in higher levels of antigen specific IgE than when administered in CFA.[14] However, the observed antigen specific serum $IgG_1$ and $IgG_{2a}$ responses were not influenced when using either alum or CFA as adjuvant. These authors suggested that primarily the type of adjuvant determined the resulting IgE production.[14] From other studies it can be concluded that the type of antigen plays, next to the type of adjuvant, a determining role in the production of other isotypes than IgE.[11–13]

The effect of alum and CFA as adjuvant on the antigen specific $IgG_1$ and $IgG_{2a}$ was studied and the total IgE antibody production after TNP–KLH immunization. Moreover, we examined the effect of continuous presence of systemic IFN-γ on the isotypes produced after immunization with TNP–KLH adsorbed on alum. Besides the effects on the primary response the influence of adjuvant on the antigen specific IgE memory formation was determined.

### Materials and Methods

*Mice:* Female BALB/c and C57BL/6 mice were bred and maintained at the Department of Immunology of the Erasmus University. All mice were aged 12–16 weeks at the start of the experiments. They were held in light-cycled rooms and had access to acidified water and pelleted food *ad libitum*. The microbiological status of the mice fulfilled the standard of 'specific pathogen free V', according to the criteria of the Dutch Veterinary Inspection, as described in the law on animal experiments. The experiments were approved by the Animal Experiments Committee of the Erasmus University.

*Immunization:* KLH (Pierce, Rockford, IL, USA) was tri-nitrophenylated to a level of 25 TNP residues per $10^5$ Da of KLH (as determined spectrophotometrically) by using tri-nitrobenzenesulfonic acid (Eastman Kodak, Rochester, NY, USA).[15] Mice were injected with 0.2 ml containing either 10 μg or 100 μg TNP–KLH adsorbed on 2 mg alum. Alternatively, either 10 μg or 100 μg TNP–KLH emulsified in 0.2 ml CFA was injected i.p., or 10 μg TNP–KLH in saline was injected every 2 days until day 14, as indicated in the results section.

*Cytokine treatment:* Mice immunized with 100 μg TNP–KLH adsorbed on alum were implanted i.p. with $2 \times 10^6$ CHO/IFN-γ cells encapsulated in alginate on days 1, 14 and 28 as described previously for CV-1/IL-4 cells.[15–17] The CHO/IFN-γ cells were a kind gift of Dr N. Arai (DNAX Research Institute, Palo Alto, CA, USA). Empty beads encapsulated in alginate were used as control for the IFN-γ treatment. No immunological effects were observed as a result of this treatment in all experiments.

*Isotype specific ELISA:* Total serum IgE, $IgG_1$ and $IgG_{2a}$ levels were measured by isotype specific ELISA as described previously.[15] Detection limits for the IgE, $IgG_1$ and $IgG_{2a}$ ELISA were 0.5 ng/ml, 0.2 ng/ml and 0.3 ng/ml, respectively. TNP specific $IgG_1$ and IgE were determined as described previously,[15] with 0.2 ng/ml and 1 ng/ml as detection limit in the ELISA, respectively. TNP–KLH specific $IgG_{2a}$ was measured by direct ELISA. Plates were coated with TNP–KLH (3 μg/ml), blocked with 1% BSA and incubated with the appropriate dilutions of serum. Subsequent steps with biotin conjugates GAM/$IgG_{2a}$ (Southern Biotechnology, Birmingham, AL, USA), SA-HRP (Jackson Immunoresearch, West Grove, PA, USA) and the substrate 2,2′-azino-bis(3-ethylbenz-thiazoline-6-sulfonic acid) (ATBS) (Sigma, St. Louis, MO, USA). Purified TNP specific $IgG_{2a}$ was used for the standard curve in this antigen specific assay. The detection limit of this ELISA was 0.4 ng/ml.

*Con A stimulation of splenocytes:* Spleen cells at $2 \times 10^6$/ml were cultured in RPMI 1640 medium supplemented with 10% heat inactivated FCS, 2 mM glutamine, 0.1 M pyruvate, 100 IU/ml penicillin, 50 μg/ml streptomycin, 50 μM 2-mercapto-ethanol in six replicate wells of a 24-well flat-bottom plate (1 ml/well) with 10 μg/ml Concanavalin A (Sigma). After 48 h culture supernatants were harvested and stored at −70°C before use.

*Determination of cytokines:* IL-4, IL-6, IL-10 an IFN-γ were determined in ELISA as described previously.[18–21] The detection limits of the ELISA were 0.2 ng/ml, 1.5 U/ml, 3 U/ml and 0.2 ng/ml, respectively.

*Statistical analysis:* Differences between groups were analyzed using Student's *t*-test. Values of $p < 0.05$ were considered significant.

### Results and discussion

Mice were immunized i.p. with 100 μg $TNP_{25}$-KLH either adsorbed on 2 mg alum or emulsified in CFA (Table 1). On the days indicated serum was collected and antigen specific $IgG_1$ and $IgG_{2a}$ and total IgE were determined in isotype specific ELISA. Serum total IgE and TNP specific $IgG_1$ and $IgG_{2a}$ peak levels were not different whether TNP–KLH was given adsorbed on alum or emulsified in CFA (Table 1). Primary immunization of BALB/c mice with 100 μg TNP–KLH either adsorbed on alum or emulsified in CFA provoked a response dominated by a 25-fold increase in TNP specific $IgG_1$ and resulting in a peak level in the serum of 1.5 mg/ml. Even a proportional higher increase was observed for TNP specific $IgG_{2a}$ upon immunization with 100 μg TNP–KLH, reaching a maximum serum level of 54 μg/ml when administered emulsified in CFA, and 78 μg/ml when adsorbed on alum (Table 1). With this does of TNP–KLH employed the primary total IgE (Table 1) and antigen specific IgE (data not shown) responses were only marginal and did not differ between the two adjuvants used. These results show that immune responses induced by TNP–KLH immunization, either adsorbed on alum or emulsified in CFA, do not differ in the isotypes and amount of antibodies produced. Immunization of C57BL/6 mice with 100 μg TNP–KLH adsorbed on alum resulted in an isotype response pattern similar to that in BALB/c mice, and also dominated by TNP specific $IgG_1$. The peak levels of the isotypes produced, however, were consistently 2-fold lower (Table 1).

It has been reported that immunization with antigen emulsified in CFA induces T cells that predominantly produce IFN-γ, and do not secrete IL-4.[6] Therefore, since IFN-γ inhibits the IL-4 induced IgE synthesis[7,8] lower levels of IgE after immunizing BALB/c mice with TNP–KLH in CFA would be expected, than when alum was used as adjuvant. Such an inhibition was not observed (Table 1), suggesting that CFA did not induce enough IFN-γ to significantly inhibit the TNP–KLH induced IgE production. Therefore, we next studied the effect of continuous presence of IFN-γ on total and antigen specific $IgG_1$, $IgG_{2a}$ and IgE synthesis induced by immunization with 100 μg TNP–KLH adsorbed on alum. To ensure a continuous systemic presence of IFN-γ CHO cells that were stably transfected with the murine IFN-γ gene and encapsulated in alginate were implanted in the peritoneal cavity of BALB/c mice 1 day before and 14 days after antigenic challenge.[15–17] Surprisingly, under these conditions IFN-γ augmented both the serum total $IgG_1$ and IgE levels, whereas it did not influence the serum total $IgG_{2a}$ levels (Table 2). However, IFN-γ treatment markedly decreased the TNP specific $IgG_1$ and IgE response, but did not influence the TNP specific $IgG_{2a}$ response, at day 14 (data not shown).

In the literature, BALB/c mice were described as IgE high-responder mice, whereas C57BL/6 mice were described as IgE low-responder mice.[22] Therefore, we next studied the effect of immunization with TNP–KLH adsorbed on alum in the presence or absence of exogenous IFN-γ on the $IgG_{2a}$ production in C57BL/6 mice. In these mice, however, immunization with TNP–KLH adsorbed on alum in the presence of exogenous IFN-γ also did not result in increased serum total $IgG_{2a}$ levels (data not shown). Nevertheless, the lower IgE production found in these mice upon TNP–KLH immunization (Table 1) suggests a lower expression level of IL-4 and/or higher level of IFN-γ, than in BALB/c mice.

The encapsulated IFN-γ producing cells secreted enough biologically active IFN-γ to enhance $IgG_{2a}$ synthesis, as was shown by implanting IFN-γ producing cells in unimmunized naive BALB/c mice. The serum total $IgG_{2a}$ levels significantly increased in

**Table 1.** Ig isotype distribution of $IgG_1$, IgE and $IgG_{2a}$ antibodies after immunization of BALB/c and C57BL/6 mice with TNP–KLH either on alum or in CFA

| Mice | Adjuvant | Isotype* | Day 0 | Day 14 |
|---|---|---|---|---|
| BALB/c | Alum | IgE | 1.6 ± 0.3 | 3.0 ± 0.5 |
| | | anti-TNP $IgG_1$ | 64 ± 14 | 1544 ± 59 |
| | | anti-TNP $IgG_{2a}$ | < 0.4 | 78 ± 15 |
| BALB/c | CFA | IgE | 1.2 ± 0.2 | 2.0 ± 0.4 |
| | | anti-TNP $IgG_1$ | 58 ± 4 | 1485 ± 68 |
| | | anti-TNP $IgG_{2a}$ | 2.0 ± 0.1 | 54 ± 9 |
| C57BL/6 | Alum | IgE | 0.1 ± 0.01 | 1.2 ± 0.4 |
| | | anti-TNP $IgG_1$ | 3 ± 0.1 | 929 ± 130 |
| | | anti-TNP $IgG_{2a}$ | < 0.4 | 19 ± 3 |

BALB/c or C57BL/6 mice were immunized i.p. either with 100 μg TNP–KLH adsorbed on alum, or with 100 μg TNP–KLH in CFA as indicated. *Total IgE and TNP specific $IgG_1$ and $IgG_{2a}$ serum levels were determined. Results are presented as arithmetic mean ± S.E.M. in μg/ml ($n = 5$).

**Table 2.** Ig isotype distribution of $IgG_1$, IgE and $IgG_{2a}$ antibodies after immunization of BALB/c mice with TNP–KLH in the presence or absence of exogenous IFN-γ

| (A) | IgE (μg/ml) | | $IgG_1$ (mg/ml) | | $IgG_{2a}$ (mg/ml) | |
|---|---|---|---|---|---|---|
| TNP–KLH/Alum | No IFN-γ | IFN-γ | No IFN-γ | IFN-γ | No IFN-γ | IFN-γ |
| Day 0 | 1.2 ± 0.3 | 1.2 ± 0.3 | 1.5 ± 0.2 | 1.5 ± 0.2 | 0.9 ± 0.1 | 0.9 ± 0.1 |
| Day 14 | 1.2 ± 0.2 | 11.4 ± 1.3 | 3.8 ± 0.2 | 3.3 ± 0.4 | 1.1 ± 0.4 | 1.2 ± 0.1 |
| Day 51 | 1.7 ± 0.3 | 61.0 ± 7.4* | 3.5 ± 0.1 | 11.7 ± 1.2* | 1.5 ± 0.1 | 1.4 ± 0.2 |

| (B) No antigen | $IgG_{2a}$ (mg/ml) | | |
|---|---|---|---|
| | Alum | Alum/IFN-γ | IFN-γ |
| Day 0 | 1.0 ± 0.1 | 1.0 ± 0.1 | 0.8 ± 0.1 |
| Day 14 | 0.7 ± 0.1 | 1.9 ± 0.3 | 1.4 ± 0.2 |
| Day 37 | 0.9 ± 0.1 | 3.0 ± 0.2** | 1.7 ± 0.3** |

BALB/c mice were injected with either 100 μg TNP–KLH adsorbed on alum (A), or alum alone (B). 2 × $10^6$ CHO/IFN-γ cells encapsulated in alginate, or empty beads encapsulated in alginate were implanted i.p. on Day –1, 14 and 28. Serum total IgE, $IgG_1$ and $IgG_{2a}$ levels were determined. Results are expressed as arithmetic mean ± S.E.M. ($n$ = 5). Statistical evaluation using Students $t$-test: *values were compared between control and IFN-γ treated mice ($p < 0.05$). **values were compared relative to day 0 ($p < 0.05$).

these mice as a result of IFN-γ treatment (Table 2). Moreover, when alum, without antigen, was given in the presence of IFN-γ, a further enhancement of $IgG_{2a}$ synthesis was observed (Table 2). Alum alone did not induce $IgG_{2a}$ production. These results suggest that an adjuvant like alum augments ongoing immune responses, in which the isotypes produced are determined by the cytokines elicited upon antigen stimulation.

Together these results suggest that upon immunization with TNP–KLH excessive IL-4 and less, or strongly inhibited levels of IFN-γ are induced. As a result, no further increase in the serum total $IgG_{2a}$ level was observed, when IFN-γ treatment was added to TNP–KLH immunization adsorbed on alum (Table 2). The unexpected effects of the IFN-γ treatment with respect to the increased serum total $IgG_1$ and IgE cannot easily be explained. It is possible that the amount of exogenously produced IFN-γ was too low to inhibit the IL-4 induced isotype switching to $IgG_1$ and IgE. These two isotypes are coupled through the process of sequential isotype switching.[23] Alternatively, IFN-γ levels could be high enough to support polyclonal bystander activation by increasing the MHC class II expression on macrophages and dendritic cells, resulting in enhanced antigen presentation.[24–26] It is known that IFN-γ decreases the IL-4 induced up-regulation of MHC class II molecules on B cells.[1] Therefore, it is possible that the observed inhibition of the antigen specific $IgG_1$ and IgE response by IFN-γ is the result of a down-regulation of MHC class II molecules on the B cells, that are essential for antigen specific T–B cell interactions.[27]

The possibility that the endogenous cytokine production profile of spleen cells had changed as a result of IFN-γ treatment was studied next. Therefore, the cytokine production by Con A stimulated spleen cells from control and IFN-γ treated mice 1 day after

**Table 3.** Cytokine production profile of splenocytes 1 day after the last of ten IFN-γ administrations

| Treatment | IL-4 (ng/ml) | IL-6 (U/ml) | IL-10 (U/ml) | IFN-γ (ng/ml) |
|---|---|---|---|---|
| Control | 0.3 ± 0.01 | 5.1 ± 1.4 | < 3 | 1.0 ± 0.1 |
| IFN-γ | 0.4 ± 0.03 | 10.5 ± 1.5* | < 3 | 0.5 ± 0.1* |

Spleen cells (2 × $10^6$/ml) pooled from either two control treated or two IFN-γ treated BALB/c mice 1 day after the last of ten control or IFN-γ administrations were cultured with Con A (10 μg/ml) for 48 h in six replicate wells. The supernatants were harvested from these wells and individually tested for cytokine production. The results are represented as arithmetic mean ± S.D. ($n$ = 6). *Statistical evaluation using Student's $t$-test $p < 0.05$.

the last control of ten control or IFN-γ administrations was determined. Similar amounts of IL-4 were detected upon culturing spleen cells from control or IFN-γ treated mice, whereas no IL-10 production could be detected (Table 3). The IL-6 production by spleen cells from IFN-γ treated mice was significantly increased when compared to the IL-6 production by spleen cells from control treated mice. However, the IFN-γ production slightly decreased, in similar culture conditions, as a result of IFN-γ treatment (Table 3), suggesting a negative feedback by IFN-γ itself. The increased IL-6 production in combination with unchanged IL-4 production suggested an increase in the number of activated macrophages and monocytes. These results substantiate the authors' opinion that IFN-γ, under the conditions used, merely acts on these types of cells.

The preferential induction of IL-4 expression *in vivo* by TNP–KLH immunization became clear by injecting BALB/c mice repeatedly with 10 μg TNP–KLH in saline, that is every 2 days until day 14. This immunization scheme resulted in 1.89 μg serum total IgE, 4-fold higher than the saline control (Fig. 1). Primary IgE production was determined because of its total dependence on IL-4.[15,28,29] Immunization

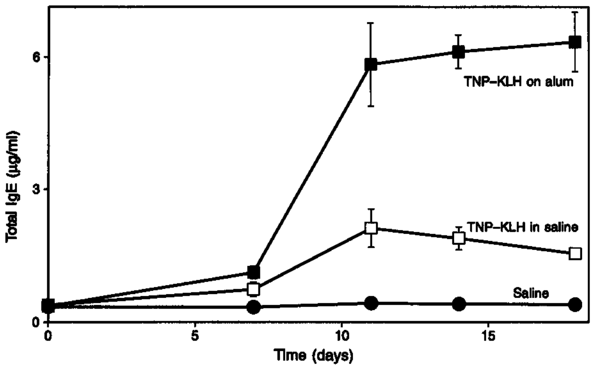

FIG 1. BALB/c mice were immunized with 10 µg TNP–KLH in saline (□) or saline alone (●) every 2 days until day 14, or immunized once with 10 µg TNP–KLH adsorbed on alum at day 0 (■) Serum total IgE levels were determined on the days indicated. Results are expressed in µg/ml as arithmetic mean ± S.E.M. ($n = 5$).

with this dose of 10 µg TNP-KLH adsorbed on alum resulted in a 20-fold increase of the serum total IgE level at day 14 (Fig. 1). These results substantiate the fact that TNP-KLH, itself, already induces IL-4 production which is reflected by elevation of the total serum IgE levels; whereas alum, used as adjuvant, merely enhances the antigen induced antibody response. Similar primary IgE responses were found upon immunization with TNP–KLH either adsorbed on alum, or in CFA (Table 1).

The effect of adjuvant on the memory formation for IgE was then studied, because it is possible that formation of memory B cells for IgE is more dependent on the ratio of IL-4 and IFN-γ than the primary IgE production. Therefore, BALB/c mice were immunized i.p. either with 10 µg TNP–KLH adsorbed on alum, or emulsified in CFA. This dose of TNP–KLH was used because it elicits a significant primary serum total IgE response (Fig. 1).[28,30] Three months later all mice were boosted with

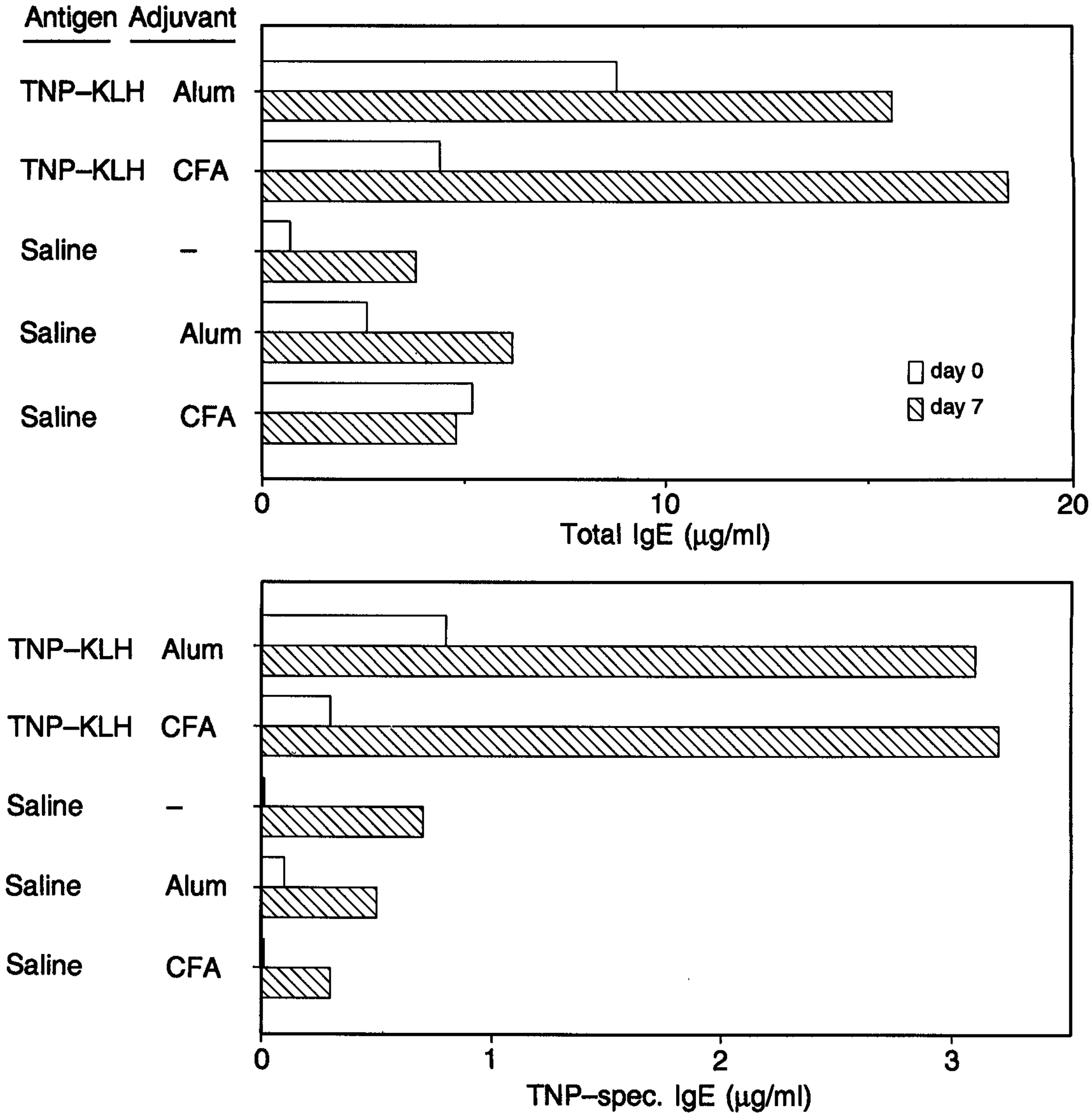

FIG. 2. BALB/c mice were primed i.p. with 10 µg TNP–KLH either adsorbed on alum or emulsified in CFA as indicated. As a control alum or CFA was injected in the absence of antigen. Three months later all mice were boosted with 10 µg TNP–KLH adsorbed on alum i.p. On day 0 and day 7 after booster total and antigen specific serum IgE levels were determined in pooled sera of four mice. Results are expressed in µg/ml as arithmetic mean of three ELISA determinations. □, day 0; ▨, day 7.

10 μg TNP–KLH adsorbed on alum. On day 7, both total and antigen specific IgE serum levels were determined in pooled sera of four mice (Fig. 2). Under these conditions it did not make any difference whether alum or CFA was used as adjuvant during priming. Upon boosting with 10 μg TNP–KLH adsorbed on alum similar levels of both antigen specific and total IgE were produced at day 7 by mice primed either with TNP–KLH adsorbed on alum or emulsified in CFA (Fig. 2). It was therefore concluded that the memory formation for IgE is also not influenced by alum and CFA.

Collectively, the results show that when using TNP–KLH as antigen, the resulting production of $IgG_1$, IgE and $IgG_{2a}$ is not dependent on the adjuvant employed. CFA and alum rather act as enhancement factors for ongoing antigen induced antibody responses, in which the carrier part of the antigen determines the isotypes produced. Upon using KLH as carrier, apparently an abundant IL-4 production is generated by carrier specific Th-2 cells which is not easily counteracted by IFN-γ. Recently, it has been described that IL-4 production is dependent on the type of antigen and the duration of antigenic stimulation.[31] It is possible that repeated antigenic stimulation of T cells, by immunization with TNP–KLH every 2 days for 14 days, induces IL-4 production resulting in IgE synthesis. When antigen is given once, the use of adjuvant could result in an antigen depot, resulting in repeated antigenic stimulation and subsequent IL-4 production. This indicates that IL-4 can be induced by antigen, independent of the type of adjuvant, resulting in an antibody response dominated by $IgG_1$.

Fox[13] has described that immunization with a protein antigen either given adsorbed on alum or emulsified in CFA induced similar T-cell proliferation and cytokine production. Moreover, in this study it was shown by limiting dilution analysis that comparable frequencies of antigen specific T cells are induced by the antigen, independent of the adjuvant used. These data support our view that it is most likely the carrier part of antigen that preferentially activates the development of either Th-1 or Th-2 effector cells, after which the adjuvant merely enhances the antibody responses generated. The possibility that other types of antigen in combination with either alum or CFA will result in different isotype patterns cannot be excluded. However, at the moment no such studies are available with respect to the type of antibodies tested in our study.

### References

1. Vitetta ES, Fernandes-Botran R, Myers D Sanders VM. Cellular interactions in the humoral immune response. *Adv Immunol* 1989; **45**: 1–105.
2. Mosmann TR, Coffman RL. Heterogeneity of cytokine secretion patterns and functions of helper T cells. *Adv Immunol* 1989; **46**: 111–147.
3. Mosmann TR, Schumacher JH, Street NF, *et al.* Diversity of cytokine synthesis and function of mouse CD4⁺ T cells. *Immunol Rev* 1991; **123**: 209–229.
4. Grun JL, Maurer PH. Different T helper subsets elicited in mice utilizing two different adjuvant vehicles: The role of endogenous interleukin 1 in proliferative responses. *Cell Immunol* 1989; **121**: 134–145.
5. Street NE, Schumacher JH, Fong TAT, Bass H, Fiorentino DF, Leverah JA, Mosmann TR. Heterogeneity of mouse helper T cells. Evidence from bulk cultures and limiting dilution cloning for precursors of Th1 and Th2 cells. *J Immunol* 1990; **144**: 1629–1639.
6. Yang X, Hayglass T. Allergen-dependent induction of interleuk-4 synthesis *in vivo*. *Immunology* 1993; **78**: 74–79.
7. Finkelman FD, Holmes J, Katona Im, *et al.* Lymphokine control of *in vivo* immunoglobulin isotype selection. *Ann Rev Immunol* 1990; **8**: 303–333.
8. Coffman RL, Lebman DA, Rothman P. Mechanism and regulation of immunoglobulin isotype switching. *Adv Immunol* 1993; **54**: 229–270.
9. Snapper CM, Paul WE. Interferon-γ and B cell stimulatory factor-1 reciprocally regulate Ig isotype production. *Science* 1987; **236**: 944–947.
10. Van Ommen R, Vredendaal AECM, Savelkoul HFJ. Secondary IgE responses *in vivo* are predominantly generated via γ,ε-double positive B cells. *Scand J Immunol* 1994; in press.
11. Kenney JS, Hughes BW, Masada MP, Allison AC. Influence of adjuvants on the quantity, affinity, isotype and epitope specificity of murine antibodies. *J Immunol Methods* 1989; **121**: 157–166.
12. Karagouni EE, Hadjipetrou-Kourounakis L. Regulation of isotype immunoglobulin production by adjuvants *in vivo*. *Scand J Immunol.* 1990; **31**: 745–754.
13. Fox BS. Antibody responses to a cytochrome c peptide do not correlate with lymphokine production patterns from helper T cell subsets. *Immunology* 1992; **75**: 164–169.
14. Beck L, Spiegelberg HL. The polyclonal and antigen-specific IgE and IgG subclass response of mice injected with ovalbumin in alum or complete freund's adjuvant. *Cell Immunol* 1989; **123**: 1–8.
15. van Ommen R, Vredendaal AECM, Savelkoul HJF. Prolonged IL-4 treatment inhibits antigen-specific $IgG_1$ and IgE formation. *Scand J Immunol* 1994; **40**: 1–9.
16. Savelkoul HFJ, Seymour BWP, Sullivan L, Coffman RL. IL-4 can correct defective IgE production in SJA/9 mice. *J Immunol* 1991; **146**: 1801–1805.
17. Savelkoul HFJ, van Ommen R, Vossen ACTM, Breedland EG, van Oudenaren A. Modulation of systemic cytokine levels by implantation of alginate encapsulated cells. *J Immunol Methods* 1994; **170**: 185–196.
18. Cherwinski HM, Schumacher JH, Brown KD, Mosmann TR. Two types of mouse helper T cell clones. III. Further differences in lymphokine synthesis between Th-1 and Th-2 clones revealed by RNA hybridization, functionally monospecific bioassays and monoclonal antibodies. *J Exp Med* 1987; **166**: 1229–1244.
19. Fletcher Starnes HJR, Pearce MK, Tewari A, Yim JH, Zou J-C, Abrams JS. Anti-IL-6 monoclonal antibodies protect against lethal *Escherichia coli* infection and lethal tumor necrosis factor-α challenge in mice. *J Immunol* 1990; **145**: 4185–4191.
20. MacNeil IA, Suda T, Moore KW, Mosmann TR, Zlotnik A. IL-10, a novel growth cofactor for mature and immature T cells. *J Immunol* 1990; **145**: 4167–4173.
21. Chatelain R, Varkila K, Coffman RL. IL-4 induces a Th-2 response in Leishmania major-infected mice. *J Immunol* 1990; **148**: 1182–1187.
22. Levine BB, Vaz NM. Effect of combinations of inbred strain, antigen, and antigen dose on immune responsiveness and reagin production in the mouse. *Int Arch Allergy.* 1970; **39**: 156–166.
23. Mandler R, Finkelman FD, Levine DA, Snapper CM. IL-4 induction of IgE class switching by lipopolysaccharide-activated murine B cells occurs predominantly through sequential switching. *J Immunol* 1993; **150**: 407–418.
24. Steeg PS, Moore RN, Johnsen HM, Oppenheim JS. Regulation of murine macrophage Ia antigen expression by a lymphokine with immune interferon activity. *J Exp Med* 1982; **156**: 1780–1793.
25. Murray HW, Spitalny GL, Nathan CF. Activation of mouse peritoneal macrophages *in vitro* and *in vivo* by interferon-γ. *J Immunol* 1985; **134**: 1619–1622.
26. Trichieri G, Perussia B. Immune interferon: a pleiotropic lymphokine with multiple effects. *Immunol Today* 1985; **6**: 131–136.
27. Parker DC. T cell-dependent B cell activation. *Ann Rev Immunol* 1993: **11**: 331–360.
28. van Ommen R, Vredendaal AECM, Savelkoul HFJ. Suppression of polyclonal and antigen-specific murine $IgG_1$ but not IgE responses by neutralizing IL-6 *in vivo*. *Eur J Immunol* 1994; **24**: 1396-1403.
29. Kuhn R, Rajewsky K. Muller W. Generation and analysis of interleukin-4 deficient mice. *Science* 1991; **254**: 707–710.
30. DeKruyff RH, Fang Y, Umetsu DT. IL-4 synthesis by *in vivo* primed keyhold limpet hemocyanin-specific CD4⁺ T cells. I. Influence of antigen concentration and antigen-presenting cell type. *J Immunol* 1992; **149**: 3468–3476.
31. Saito S, Dorf ME, Watanabe N, Tadakuma T. Preferential induction of IL-4 is determined by the type and duration of antigenic stimulation. *Cell Immunol* 1994; **153**: 1–8.

ACKNOWLEDGEMENTS. We thank Mr T. M. van Os for graphic design, Mr J. Brandenburg and Ms A. van't Hof for animal care and Prof. Dr R Benner for reading the manuscript critically. This work was supported by the Netherlands Foundation of Medical Research (NWO).

**Received 16 May 1994; accepted in revised form 22 June 1994**
